# The Potential to Address Disease Vectors in Favelas in Brazil Using Sustainable Drainage Systems: Zika, Drainage and Greywater Management

**DOI:** 10.3390/ijerph19052860

**Published:** 2022-03-01

**Authors:** Susanne M. Charlesworth, Debora C. Kligerman, Matthew Blackett, Frank Warwick

**Affiliations:** 1 Centre for Agroecology, Water and Resilience, Coventry University, Coventry CV8 3LG, UK; 2 Fundação Oswaldo Cruz, FIOCRUZ, Departamento de Saneamento e Saúde Ambiental (DSSA) (ENSP), Rio de Janeiro 21040-900, Brazil; deboracyklig@gmail.com; 3 School of Energy, Construction and Environment, Faculty of Engineering, Environment and Computing, Coventry University, Coventry CV1 5FB, UK; aa8533@coventry.ac.uk (M.B.); aa4510@coventry.ac.uk (F.W.)

**Keywords:** favela, Zika, greywater, sustainable drainage systems (SuDS), Brazil

## Abstract

Residents of informal settlements, the world over, suffer consequences due to the lack of drainage and greywater management, impacting human and environmental health. In Brazil, the presence of the *Aedes aegypti* mosquito in urban areas promotes infections of the Zika virus as well as companion viruses, such as dengue, chikungunya and yellow fever. By using observation and interviews with the community, this paper shows how a simple sustainable drainage system approach could prevent the accumulation of on-street standing water, and thus reduce opportunities for the mosquito to breed and reduce infection rates. During the interview phase, it became apparent that underlying misinformation and misunderstandings prevail related to existing environmental conditions in favelas and the role of the mosquito in infecting residents. This inhibits recommendations made by professionals to reduce breeding opportunities for the disease vector. Whilst unrest is an issue in favelas, it is not the only issue preventing the human right to reliable, safe sanitation, including drainage. In “pacified” favelas which may be considered safe(r), the infrastructure is still poor and is not connected to the city-wide sanitation/treatment networks.

## 1. Introduction

**Figure 1:**
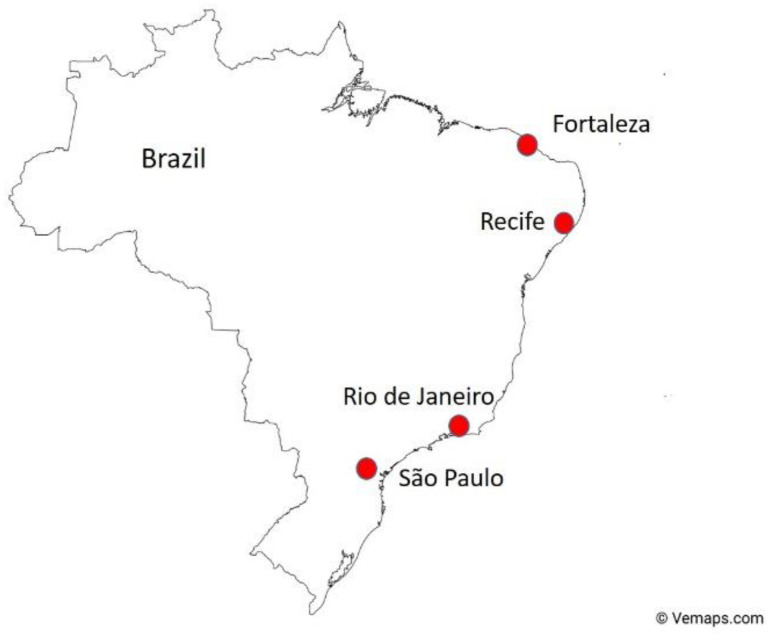
Location of the highest incidences of ZIKV and microcephaly cases in Brazil (Brazil Map by Vemaps.com, accessed on 20 January 2022).

This paper is based on a British Council Newton Institutional Links Award between the UK and Brazil, focused on reducing the incidence of infection of the Zika virus (ZIKV), mainly transmitted by the bite of the female *Aedes aegypti* mosquito. ZIKV has potentially devastating effects on unborn children, with a variety of congenital and developmental conditions, including microcephaly, which, in the worst cases, substantially impacts their development. Whilst found in 94% of the country, the highest density of ZIKV infections and microcephaly cases were found mainly in Brazil’s most populated areas, in the northeast and southeast of the country, including Fortaleza, Recife, Rio de Janeiro and São Paulo (see Figure 1; [1]); areas typically associated with many informal settlements.

There are a variety of terminologies used to describe and define informal settlements; in Brazil, favela or subnormal agglomeration are generally used, the latter introduced by [2], whereby the settlement has the following characteristics:

1. An illegal occupation, construction of which took place on land belonging to others; or where title to the land was established in the last 10 years;
2. Includes one, or both of the following:
  - Construction did not follow municipal regulations, with narrow and uneven roads, parcels of land uneven in shape and/or size; development not supervised by regulatory agencies;
  - Lack of public services.

*Favela* is generally used to identify an informal settlement in the Brazilian context; thus this will be used hereinafter.

It was suggested by [3,4] that there may be a correlation between ZIKV epidemiology and conditions in favelas due to their higher confirmed ZIKV and microcephaly cases, in comparison with elsewhere in the urban environment. Favelas often lack adequate sanitation infrastructure that safely manages stormwater (or water running off surfaces due to heavy rainfall) or greywater (wastewater generated in the household due to activities, such as personal bathing, preparation of vegetables and clothes washing, but not water that goes down the toilet or is associated with human bodily waste). Instead, it is often disposed of via open drainage ditches, a local water body or simply on the street, leading to standing water between dwellings, hence providing suitable breeding environments for ZIKV vector mosquitoes, mainly *Aedes aegypti* [5]. However, any open area with standing or stagnant water, or collecting in solid waste, such as discarded food packaging, car tyres, water storage containers, blocked gutters on buildings, plant pots/leaves can also provide opportunities for the mosquito to breed [3,6].

Accumulation of uncollected solid waste can also block drains, pipes and ditches, effectively reducing the capacity of existing drainage infrastructure [7]. Its accumulation encourages the pooling of stormwater, risking human and environmental health and attracts mosquitoes, flies, ticks and rats, leading to incidences of Lyme Disease, Weil’s disease and malaria [8,9,10,11,12]. At the same time, the provision of a reliable potable supply would prevent the storage of drinking water in open containers in the household. In the short term, this can be addressed by the provision of sealed containers to prevent vector ingress and drinking water contamination. However, despite infrequent potable water supplies and inadequate surface water management in favelas, these issues were not identified as potential problems in managing the ZIKV outbreak [13].

Conventional mosquito-vector control methods, including the use of insecticides and larvicides, are useful at the small, street and individual dwelling scale, but urbanisation and the associated increase in population require larger scale, longer-term approaches [14]. There is also evidence that the widespread and continual use of these compounds has a detrimental impact on human health [15] documenting negative developmental effects on infants. It is also well known that the mosquito may develop resistance, reducing the effectiveness of these measures over time [16]. Longer-term changes could thus include modifying housing design and characteristics, screening doors and windows [17], preventing the accumulation of water in open areas and the provision of reliable piped water supplies [6]. Introduction of such approaches could support the aims of the UN Sustainable Development Goals (SDG), particularly SDG6’s ‘Clean water and sanitation’ [5,6,14,18]. However, in information about both the SDGs and the United Nations Universal Declaration of Human Rights (United Nations General Assembly, Resolution 64/292, 2010), the emphasis has been on supplying potable water and sanitation, with the latter specifically focusing on the provision of toilets and washing facilities. Whilst “sanitation” does implicitly include drainage, nonetheless, neither it nor wastewater management is explicitly mentioned [5], and in fact, in the 2018 SDG6 report [19], of the 199 pages, there is little mention of anything related to sustainable drainage approaches. On p. 21, for example, the report states:

“Interest is growing in nature-based solutions (NBSs), which use or mimic natural processes to increase water availability (e.g., soil moisture retention and groundwater recharge), improve water quality (e.g., natural and constructed wetlands and riparian buffer strips), and reduce water-related risks by restoring flood plains and constructing decentralized water retention systems such as green roofs.”

However, further into the document (p. 146), it admits:

“… despite a long history and growing experience in the application of NBSs, there are still many cases where water resources policy and management ignore NBS options—even where they are obvious and proven and effective. Water management remains heavily dominated by traditional, human-built (grey) infrastructure, and the potential for NBSs remains underutilized. Evidence suggests that this is still well below 5% of the total investment in water resources management infrastructure.”

Importantly on p. 136:

“Wastewater and inadequate drainage provide ideal breeding grounds for mosquitoes (known as disease vectors), which transmit malaria, dengue, chikungunya and zika.”

There is, therefore, a strong case to be made for drainage to be given more emphasis, and its use encouraged, in particular Sustainable Drainage Systems (SuDS) or NBS, which can manage both surface and wastewater cost-effectively and efficiently without recourse to chemical treatment [5,8,20]. At the very least, by allowing such water to infiltrate under the surface, it renders it unavailable for disease vectors, such as mosquitoes to breed in. However, this can negatively impact groundwater quality with contaminants, including organic matter, micro-pollutants and pathogens; methods to infiltrate the water, therefore, need to be designed properly [21,22].

Thus, the proposal is that due to poor sanitation and drainage, favelas provide the greatest opportunities for mosquitoes to breed, leading to higher infection rates for ZIKV and companion viruses, such as chikungunya, dengue and yellow fever, enabling their co-management.

The aims of this paper are therefore three-fold:

1. To examine any relationship between incidences of ZIKV infection and favelas;
2. To investigate whether there is a connection between lack of drainage and incidences of ZIKV infection;
3. To assess drainage and greywater management provision in favelas.

The methods were therefore developed to facilitate the collection of data in order to address the three aims given above, including selection of sites where the study was based, the recruitment of participants, and analytical methodology. The results and discussion provide examples from the interviews which contextualise the study in terms of the favela communities, the key points from which summarise the project outcomes. This paper, therefore, presents new information on the potential to use a simple, sustainable approach to drainage, which has not been considered before in the challenging environments of favelas in Brazil. This approach could have far-reaching benefits, including preventing the accumulation of runoff between dwellings and denying access to the ZIKV-carrying mosquito.

## 2. Methods

The study overall adopted a mixed-methods approach utilising two strategies:

1. Observation;
2. Key informant interviews.

This paper reports on the outcomes of both strategies.

### 2.1. Site Selection

Figure 1 shows the locations of areas with the highest concentrations of ZIKV and microcephaly cases in the northeast and southeast of Brazil. Fortaleza and Rio de Janeiro were chosen because of their location, the availability of primary data, and because contact had been made with local government organisations and community leaders.

The state capital of Ceará, Fortaleza is the fifth largest city in Brazil with a population estimated at 2.7 million in 2021 (IBGE Census: https://www.ibge.gov.br/cidades-e-estados/ce/fortaleza.html, accessed on 20 January 2022). The favelas house 16% of the population, totalling nearly 0.4 million people, living in 509 communities [23].

The Metropolitan Area of Rio de Janeiro has a total population of 13.544 million [24]. There are a total of 2990 favelas in Rio de Janeiro [25,26], and in common with Fortaleza, challenges include inadequate housing and the poor management of water resources, sanitation and drainage—which contribute to many environmental and health issues, one of which is that the conditions provide suitable environments for mosquitoes to breed [27].

### 2.2. Observation

Preliminary observations were made of the management of surface water and wastewater in two favelas in Fortaleza, one in Maracanaú to the southwest of the city, and Rosalina to the south. One was visited to the north of Rio de Janeiro, Ouro Preto, which included more traditional drainage systems.

### 2.3. Data Collection

Collection of primary data in Rio de Janeiro focused on interviews and the selection of key informants; whilst Rio is not Brazil’s capital nonetheless it is the location of many government bodies, public institutions and Non-Governmental Organisations. One of the main lessons learnt from the design and construction of a SuDS demonstration area in the Gawilan refugee camp, Kurdistan Region of Iraq [28], was that engagement with key stakeholders is vital to enable schemes to be built in the first place, but also to ensure that they achieve longevity and the desired outcomes. In-depth, semi-structured interviews were therefore carried out with members of the favela community, external stakeholders (health workers and government representatives) and academics (see Table 1) in Rio de Janeiro to establish their views regarding drainage and greywater management provision, and the potential for SuDS to address the issue of ZIKV infection in favelas. Thus, participants from diverse backgrounds, credible in their own fields, with the potential to influence policymaking, formed the basis of the contacts able to provide an appraisal of the potential to use SuDS to address disease vectors in favelas.

#### 2.3.1. Recruiting and Interviewing Participants

Participants were recruited using snowballing [29,30] and purposeful sampling methods [31,32]; they included known Brazilian academics who were contacted by email and supplied with the intended aim, objectives and data collection methods for the project. Informants then distributed the details of the project amongst their own networks and, if they were interested, they subsequently made contact with the researcher, either directly or via their informant. Sixteen in-depth face-to-face interviews were conducted in total; as shown in Table 1, their backgrounds included a broad range of expertise and experience.

Two complimentary interview schedules were developed based on the four foci of the project around governance, favelas, ZIKV and sanitation. These were designed to account for the contrasting backgrounds of participants, which included the favela community members themselves, although civil unrest connected with the recent election results at the time of fieldwork made making contacts difficult.

During all interviews, a translator was present for the duration of the process. Each question was carefully structured for ease of translation and to ensure optimal data could be obtained from each respondent. The interviews were semi-structured in approach with only a provisional order of questions to enable participants to lead the conversation based on their own knowledge, promoting additional lines of questioning that further explored any other points of interest. This was facilitated by the translator directly or with simultaneous translation between Portuguese and English [33,34]. This was a carefully considered factor to ensure potential language barriers were addressed and to ensure the comfort of each participant. The breadth of respondents enabled an understanding of key factors, such as government policy as well as individual perspectives of the challenges of living with inadequate infrastructure and poor environmental health in favelas.

#### 2.3.2. Analysis

**Figure 2:**
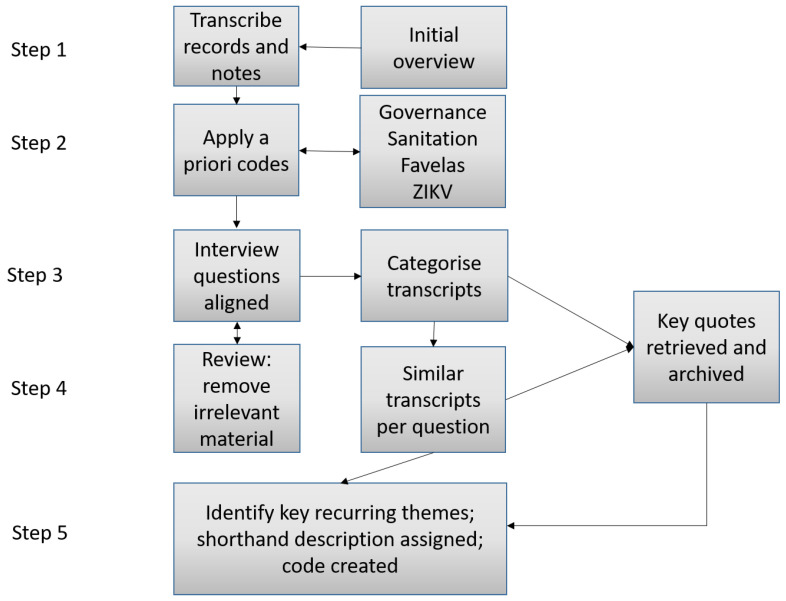
Steps followed in Template Analysis to analyse results of interviews.

To generate results following analysis of the face-to-face interviews, a Template Analysis approach was used [35] as shown in Figure 2. Coding was supported by the application of relevant codes, which were related to the four themes underpinning the conceptual framework of the project: Governance, Sanitation, ZIKV and Favela(s). Coding was carried out by hand using Microsoft Office Word and Excel to support the notation of codes [36]. Once all transcripts and questions had been reviewed, key quotations were compiled into a codebook to enable further analysis of the dataset [37]. The steps that were followed are summarised in Figure 2. Under Step 4, material that was considered irrelevant, such as comments not related to the purpose of the interviews, was discarded.

## 3. Results and Discussion

### 3.1. Observation: Opportunities to Site SuDS to Address Standing Surface Water

**Figure 3:**
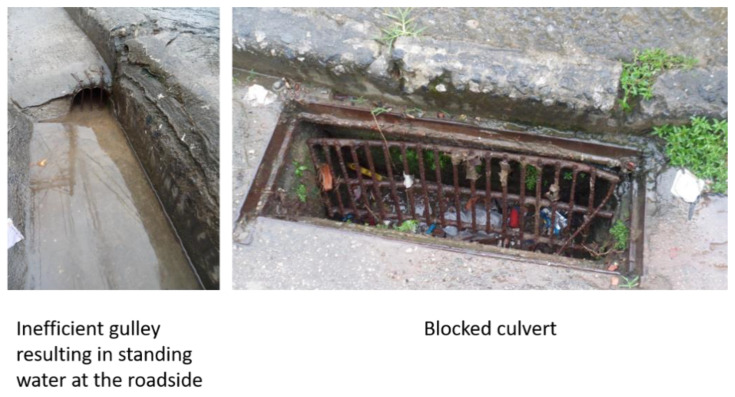
Conventional drainage, Ouro Preto, blocked with solid waste.

The conventional drainage observed in Ouro Preto suffered from lack of maintenance, as blockages resulted in standing water with no option for conveyance or infiltration (Figure 3).

**Figure 4:**
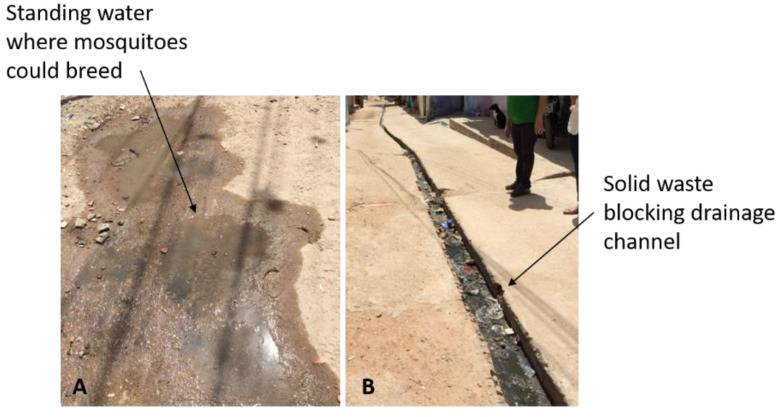
(**A**,**B**) Blocked and inadequate drainage, with mosquito larvae in standing water outside dwellings in favelas, Fortaleza, Brazil.

Overall, it was found that drainage was inadequate at best (see [5] and Figure 4A,B). Figure 4A shows standing water outside a dwelling in which many mosquito larvae were observed, showing the potential for disease vectors to proliferate under such conditions. Problems associated with blockages due to solid waste were also observed, with undersized and open drainage channels (Figure 4B) and little or no provision made for the disposal of household greywater.

**Figure 5:**
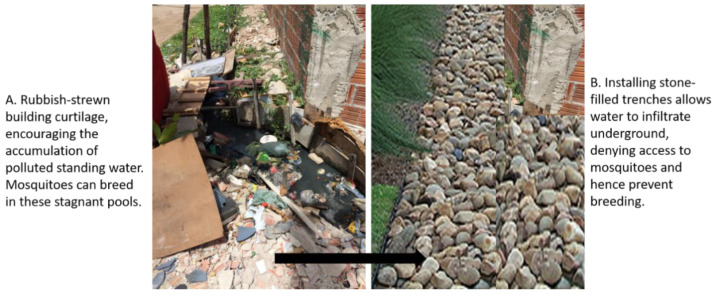
(**A**) Accumulation of standing water in solid waste around a favela dwelling; installing a stone-filled trench; (**B**) has the potential to deny mosquitoes access to water in which they could breed.

Figure 5A shows standing water observed around a favela dwelling in Fortaleza. This ponding was significant in some of the areas visited, highlighting it as a major issue. If this water could be encouraged to infiltrate into the ground, or a structure installed which could make this possible, then a substantial breeding ground for mosquitoes would be removed. The issue of solid waste would need to be tackled as a matter of urgency, but as is shown in Figure 5B, a simple stone-filled trench could deny access to the standing water, and hence reduce mosquito breeding in that area. Obviously, ground conditions would need to be investigated properly for the trench to efficiently perform its function, such that slopes, soil type, infiltration capacity, etc., would need to be determined. A maintenance strategy would also need to be put in place, even if it was just solid waste removal it would help performance. Whether at the community level or with local or regional government support, there would also need to be financial assistance. Further details can be found in country-specific guidelines, where available, such as [38] in the UK.

### 3.2. Interviews: Public Health Awareness Education and the Connection between Favelas and ZIKV

**Figure 6:**
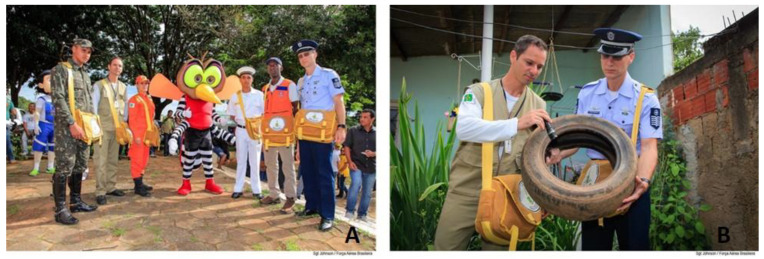
The Brazilian Air Force assisting in an exercise “all against Zika” in the local community, checking tyres containing water. (**A**) “Todos contra o Zika” by Força Aérea Brasileira—Página Oficial is licenced under CC BY-NC-SA 2.0. (**B**) “FAB participa de ação de combate aos criadouros de mosquitos” (Brazilian Air Force (FAB) participates in actions against mosquito breeding sites) by Força Aérea Brasileira—Página Oficial is licenced under CC BY-NC-SA 2.0.

Efforts had been made throughout urban areas to encourage all residents to check their local environment for standing water to reduce the incidence of mosquitoes (Figure 6). Leaflets were distributed, advice shared during door-to-door visits and free insect repellent given to pregnant women, to minimise the chance of contracting ZIKV. However, in spite of these attempts at engagement, one of the participants was frustrated by the lack of uptake of these measures in both formal and informal (favela) communities:

“some have taken precautions, but most have not. I will not give up; every time I go into the community, I continue to give out information”.

Participants, including both favela residents and the academic community, felt that the information that was in circulation was misleading or ambiguous, in particular about the ZIKV and also the breeding habits of the *Aedes aegypti* mosquito, stating that for instance:

“The vector of Zika … needs potable water to reproduce in.”

“The attention of the media and public was around sensitisation towards clean water management, not greywater or sewage.”

This lack of knowledge and awareness due to ineffective government public health messaging is encapsulated in one participant’s statement that:

“many people [did] not trust the link between Zika and microcephaly” and that there were.

“concerns that microcephaly is linked to poisoning from insecticides and not the mosquito at all.”

After issues associated with sanitation, disposal of solid waste was a problem that was mentioned most frequently. The main reason stated was that dumpsters were not provided in favelas, so it was typical for rubbish to be thrown into the street. Even in the rare cases where dumpsters were provided, there was no system for them to be emptied. Thus:

“If you have rubbish in the house… or just throw it outside… when it rains this then becomes a breeding point for mosquitoes … I do not think they follow my instructions [as a community health worker] to put their solid waste in a nearby dumpster.”

In terms of the connection between incidences of ZIKV and the favelas, half of the participants disputed this, with one commenting that:

“the territory of Zika is all over the city.”

However, there was agreement that there appeared to be:

“higher levels of microcephaly in favelas.”

In Rio de Janeiro specifically, a relatively high proportion of ZIKV cases were recorded in formal, wealthier communities with adequate sanitation, reliable water supplies and waste disposal.

“the areas we found the highest rate of cases were not … the poorest.”

In a review of the incidence of dengue and whether it is a disease just associated with poverty, [39] found that this companion virus to Zika would become increasingly significant for those they termed the “non-poor”, supporting statements from the present study, such as:

“In Rio [de Janeiro] it is often hard to differentiate between favela communities and rich areas. This is because … there is less definition between rich and poor areas … they live side-by-side.”

(see [40])

Some of the reasons for the incidences of ZIKV in the wealthier areas were possibly due to households:

“[failing to] cover swimming pools … [and] remove empty plant pots”; “apartment roof slabs … allow[ing] water to pool inside … [that then go unchecked] for weeks”; “gutters not regularly cleaned”; “balconies left to store water on.”

Some of these issues were due to the apartments that families only

“visit at weekends and holidays.”

This has led to the necessity for specialist Community Surveillance Agents having to visit these apartments in order to:

“apply insecticide, empty swimming pools, and cover breeding sites.”

**Figure 7:**
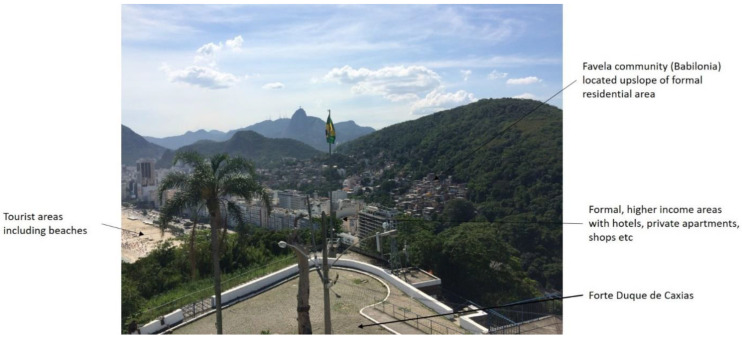
Location of some of Rio de Janeiro favelas.

The connection between incidences of ZIKV and their spatial prevalence is therefore complex in Rio de Janeiro. This is exacerbated by the topography, whereby the favelas are located upslope of the apartments, hotels and beaches leading to storm and grey water flowing down through these areas potentially carrying the mosquitoes with it (see Figure 7). 

### 3.3. Key Informant Interviews: Attitudes to Drainage

The main findings were that:

- Whilst it was felt that drainage was an important component in sanitation and water management, it needed to be integrated into a holistic environmental health management approach to include disposal of solid waste, incorporation of potable water supplies, provision of suitable sanitation, with recognition of the roles of health and hygiene in an overall strategy. Studies by [7,41], found that flooding or ponding issues were not considered to be a priority by communities since they rarely connected drainage problems with other challenges. Parkinson (2003) did find that residents of informal settlements identified flooding as something that happened frequently, with associated damage and inconvenience.
- Whatever approach was used to reduce the breeding sites for the ZIKV-carrying mosquito, it needed to be introduced and integrated throughout the whole urban area, in a joined-up approach, and not just applied to informal neighbourhoods.
- For those professionals working in favelas, their first priority was increased awareness-raising/education around aspects of environmental and public health in urban areas. This had to be addressed before any infrastructure improvement, including drainage.
- There was a lack of understanding of the lifecycle of *Aedes aegypti* and ZIKV itself, even amongst participants from all backgrounds interviewed for the research. This reflects a lack of connection with suggested prevention strategies (and a failure of public health messaging), such as that between standing water, mosquito breeding and subsequent infection with ZIKV.
- It was felt that there was not enough strong evidence demonstrating the incidence of ZIKV infection focused just on favelas. This is evidenced by ZIKV and microcephaly cases also occurring in wealthy areas, where there may be better awareness of such issues [42,43,44].

Taken in isolation, there is promise in using a SuDS approach, and in interviews, reference was made to Nature-Based Solutions and green roofs. However, whatever the potential solution might be, it would need government and community willingness and support to be enacted. Furthermore, whilst it is claimed that the “Unidades de Policia Pacificadora” (UPP; Peacekeeping Police Units) programme introduced in Rio de Janeiro in 2008 reduced unrest in the favelas [45], prejudice against favela communities linked to drug-trafficking and crime is still common [46].

According to [47], “internal and external stakeholders hold negative perceptions of each other” which leads to certain activities they could have engaged in together being prevented. An example of this is “slum upgrading” and activities around infrastructure improvements at the household level [48,49,50]. During High Court investigations related to corruption and major sporting events, government budgets and agendas were disrupted, and as a result, there have been few attempts at re-establishing any infrastructure construction projects, for example, the Program of Acceleration of Growth-Slum Upgrading (PAC-UAP); [51]). Lesser public health-related programmes continued, for example, with the training of community health workers living in the favelas. This initiative bypassed any need for local government agents from having to enter the favelas in order to engage with the community. Currently, therefore, whilst there is some interest in improving water management in favelas, the environment is not stable enough. Whilst a respondent stated, “Sanitation must be everywhere” and as stated above “the territory of Zika is all over the city” unfortunately, any water management initiatives have to come from the community itself, since there is a lack of interest in improving the lives, in general, for those who live in informal settlements, whichever the country. However, communities should not be left to resolve these problems; governments need to be held accountable for their responsibility to provide equitable and safely managed Water, Sanitation and Hygiene infrastructure and services for all, which includes the informal sector.

## 4. Conclusions

Observations have identified the potential to use SuDS in favelas to specifically target issues related to the lack of drainage or greywater management by installing structures to encourage infiltration of contaminated surface water into the ground, thus reducing the opportunity and locations for mosquitoes to breed. In high-income countries, the design and implementation of SuDS is reasonably straightforward; however, the same cannot be said for low-income countries where drainage infrastructure is either insufficient or totally lacking due to disengaged governance/accountability. When added to the potential impacts of climate change, increasing urbanisation and poor or non-existent planning regulations at the city scale, SuDS alone would not be able to address all of these challenges. Using SuDs can only be successful where urban policy and public-private partnerships can work together with adequate resources and funds to ensure completion and longevity of the strategy employed. The interviews revealed a lack of awareness of alternative drainage options (such as SuDS) for most of the participants, although as indicated in Section 3.3, NBS, infiltration measures and the use of green roofs were mentioned. There was also a lack of understanding of the connection between drainage, greywater management and the ability of mosquitoes to breed and potentially pass on the ZIKV to their human hosts.

It is thus very early for Brazil to be considering the use of SuDS, particularly associated with the favelas. Nonetheless, there have been initiatives at the community level, as well as academic examples of the ability to use SuDS to address drainage challenges, for example, as was shown in some refugee camps in the Kurdistan Region of Iraq [28] as well as informal settlements. These do show potential, but this also highlights the urgency of financial investment and recommendations for informal communities, where there are issues related to sewage, greywater and solid waste management, exacerbated by population density, the lack of infrastructure and land tenure. Issues with the lack of water and sanitation services are encapsulated in [52], who highlight the role that regulation could play in addressing inequality of service provision by prioritising the poor. However, as they state, neither the national government nor regulatory agencies have supported inclusiveness, in spite of projects, such as the National Water Agency of Brazil (ANA) or the Brazilian Development Bank (BNDES), which the federal government supported; in fact, “They are nobody’s land” “are excluded” and “not included in the statistics”. So, whilst the importance of regulation is acknowledged, currently, these problems lead to unsatisfactory disposal practices, which encourage the proliferation of vector-borne diseases.

Based on these conclusions, future lines of research which could be considered include the mapping of mosquito-vectored occurrences of ZIKV and associated congenital microcephaly cases, the provision of secure and safe potable water supplies in the favelas, and the presence or absence of adequate drainage and sanitation. Drainage and sanitation-related data are available from Brazil’s census database, the National Institute of Geography and Statistics (Instituto Brasileiro de Geografia e Estatistica, IBGE) [53], with associated metadata from [2] with drainage and sanitation conditions derived from Marques (2007). It is vital that the community and local authorities are involved, and thus before any recommendations can be formulated, results and outcomes of the project would need to be disseminated widely to enable an assessment to be made of the level of interest. This would include an evaluation of the multiple benefits of the approach including environmental improvements, which could address quality of life for the residents. These strategies would finally include co-production of a design whose recommendations would include the production of guidelines on the construction, maintenance and operation of SuDS in favelas.

To address these multiple issues, implementing SuDS in favelas could be considered due to their adaptability and scalability. The reduction in standing water would have positive impacts on environmental and human health in reducing transmission of a wide range of waterborne (e.g., cholera, typhoid), water-based (e.g., schistosomiasis) and water-vectored (e.g., ZIKV, dengue and malaria) diseases [54]. In terms of wider environmental improvements, river contamination by polluted runoff or direct discharge would reduce, specifically improving water quality, and the aquatic environment in general. Innovations such as these have the capacity to create opportunities for cities with inadequate drainage systems to leapfrog intermediary steps to become more resilient and sustainable. They also have substantial potential to be applicable worldwide, in different contexts and climates [55], and improve the ability to achieve the SDGs, in particular SDG6.

## Figures and Tables

**Table 1:** Interviewees in the project, and an indication of specialisation areas only, in order to preserve anonymity.

Background of Participant	N° of Participants
**Social Science**	
Analyst: Health Management	1
Researcher	2
**Natural Science**	
Technologist	2
Subject specialist	1
Sanitary Engineer	1
Researcher	2
Laboratory Technician	1
**Health Workers**	
Community Health	1
Nurse	1
**Government Employees**	
Subject specialist	1
Technician	1
**Favela Community**	2
**Total**	16 (9 women/7 men)
